# Surveillance of Outbreaks of SARS-CoV-2 Infections at School in the Veneto Region: Methods and Results of the Public Health Response during the Second and Third Waves of the Pandemic between January and June 2021

**DOI:** 10.3390/ijerph182212165

**Published:** 2021-11-19

**Authors:** Michele Tonon, Filippo Da Re, Chiara Zampieri, Michele Nicoletti, Riccardo Caberlotto, Francesco Paolo De Siena, Gaia Lattavo, Anil Minnicelli, Alberto Zardetto, Benedetta Sforzi, Elisa Ros, Michele Mongillo, Alessandro Scatto, Elena Vecchiato, Vincenzo Baldo, Silvia Cocchio, Francesca Russo

**Affiliations:** 1 Regional Directorate of Prevention, Food Safety, Veterinary Public Health—Regione del Veneto, 30123 Venice, Italy; michele.tonon@regione.veneto.it (M.T.); filippo.dare@regione.veneto.it (F.D.R.); elisa.ros@regione.veneto.it (E.R.); michele.mongillo@regione.veneto.it (M.M.); francesca.russo@regione.veneto.it (F.R.); 2 Department of Cardiac, Thoracic and Vascular Sciences and Public Health, University of Padua, 35122 Padova, Italy; chiara.zampieri.8@studenti.unipd.it (C.Z.); michele.nicoletti@studenti.unipd.it (M.N.); riccardo.caberlotto@studenti.unipd.it (R.C.); francescopaolo.desiena@studenti.unipd.it (F.P.D.S.); gaia.lattavo@studenti.unipd.it (G.L.); anil.minnicelli@studenti.unipd.it (A.M.); alberto.zardetto@studenti.unipd.it (A.Z.); benedetta.sforzi@studenti.unipd.it (B.S.); silvia.cocchio@unipd.it (S.C.); 3 Informative Systems Unit, Azienda Zero—Regione del Veneto, 35131 Padova, Italy; alessandro.scatto@azero.veneto.it (A.S.); elena.vecchiato@azero.veneto.it (E.V.)

**Keywords:** COVID-19, school, Italy, surveillance, public health, epidemiology

## Abstract

During the COVID-19 pandemic, many countries adopted various non-pharmacological interventions to contain the number of infections. The most often used policy was school closures. We describe the strategy adopted by the Veneto Regional Authority to contain transmission in school settings. This included a detailed school surveillance system, strict contact tracing, and maintaining school attendance with self-monitoring for symptoms whenever possible. All analyzed COVID-19 cases among children, adolescents (0–19 years old), and school staff were registered using a web-based application between 4 January 2021 and 13 June 2021. During the study period, 6272 episodes of infection in schools were identified; 87% were linked to a student index case and 13% to school staff; 69% generated no secondary cases; 24% generated one or two; and only 7% caused more than two. Our data may help to clarify the role of school closures, providing useful input for decisions in the months to come. Good practice in public health management needs tools that provide a real-time interpretation of phenomena like COVID-19 outbreaks. The proposed measures should be easy to adopt and accessible to policymakers.

## 1. Introduction

In response to the COVID-19 pandemic, school closures were imposed worldwide to limit the transmission of SARS-CoV-2 infection. The rationale for such a non-pharmacological intervention (NPI) was based on experience with other respiratory viruses, such as influenza, which children have a substantial role in transmitting [1]. In general, NPIs may be public health measures to reduce the spread of infections in the absence of vaccines and effective treatments. In the case of SARS-CoV-2, the Italian government limited citizens’ movements around the country, and drew up a list of essential workers who continued to work while all other activities were suspended. The NPIs adopted also included early case isolation, social distancing, using face masks, and closing schools and businesses. These strategies limited virus-related mortality, and prevented local healthcare systems from being overwhelmed [2,3].

During the early stages of the pandemic, closing schools was one of the NPIs most widely used around the world, though its real efficacy in mitigating coronavirus outbreaks was unclear. Many countries, including Italy, initially chose to close kindergartens, elementary, middle, and high schools, and universities nationwide. General hygienic and sanitary measures would have been difficult to apply in these settings, due to the number and age of the school population (it is impossible, for instance, to ensure younger children’s proper and continuous use of face masks).

Between March and April 2020, at least 192 countries introduced school closures, which affected more than 90% of the world’s student population. Later, in August 2020, the European Centre for Disease Prevention and Control (ECDC) reported that, from the available data, school closures seemed unlikely to be effective in reducing the community transmission of COVID-19 unless the closures were part of a systematic national strategy [4]. A subsequent update of this document confirmed that the decision to close schools should be used as a last resort, given the negative physical, mental, and educational impact on children, and the economic effects on society [5]. The ECDC’s report on the limited benefit of school closures described it as necessary only in the case of a massive spread of the infection among the population. The role of younger people as vectors of the infection was not well known, and the limited data available varied (also depending on how accurately asymptomatic cases were identified) [6]. Meanwhile, the educational impact of school closures on children, and the economic effects on families, were significant [7,8].

Governments consequently introduced various measures to reopen schools safely and keep them open. These measures were based on three broad intervention categories to detect and contain the transmission of SARS-CoV-2: organizational measures, structural and environmental measures, and surveillance and response measures [9].

After the first period of almost total school closure, the Italian government opted to reopen schools with a dedicated plan: 2.4 million single-user desks were delivered directly to schools; 170,000 L of hand sanitizer were distributed weekly; gatherings at school entrances and exits were minimized by creating temporal and spatial pathways for accessing school spaces; mixing of classes during curricular activities was forbidden; all extra-curricular activities were suspended; if classrooms could not ensure social distancing, students were divided into two groups that alternately attended face-to-face teaching at school or continued with remote learning at home [10].

To help keep the school environment safe, the Italian Superior Institute of Health produced a position paper, immediately adopted by the government, stating that school transport should be no more than 80% full [10,11]. The Italian Ministry of Education also published a list of new safety protocols for educational settings. All students over the age of six were required to wear face masks while moving around school buildings, but not while they sat at their desks and stayed one meter away from each other. Teachers and all other school staff had to wear face masks inside schools.

With rising infection rates in mid-October 2020, during Italy’s so-called “second COVID-19 wave”, more restrictive measures were adopted. The Italian Ministry of Education suspended attendance at high schools, and remote learning started again nationwide. This provision remained in place until the end of January, when 50–75% of in-person attendance was allowed to resume.

With the start of a new academic year in autumn 2021, an evidence-based update on the use of NPIs was called for. To reassess the situation, other factors needed to be analyzed, such as the timing and duration of previous school closures, and the types of school or school grades that were closed. A detailed account of the impact of schools on the spread of the COVID-19 virus has yet to be published. The add-on effect of closing schools along with other NPIs adopted has yet to be examined too, even after 15 months of pandemic (partly because of the risk of confounders and collinearity issues) [12]. Mathematical models are the only approaches that have been used, but their usefulness depends on local social behavior [13].

Given the above considerations, the objectives of this paper are: (I) to describe the impact of a centralized school surveillance system on public health strategies during the second and third waves of the COVID-19 pandemic in Italy (January to June 2021); (II) to describe the contact-tracing strategies implemented in schools and the results recorded; and (III) to produce new scientific evidence to support better social health policies.

## 2. Materials and Methods

The study began on 4 January 2021, and the first day of lessons after the Christmas break was 7 January 2021. The academic year ended on 8 June 2021, but we ended the surveillance on 13 June 2021 to include the last asymptomatic infections. During the study period, Italy recorded second and third peaks of contagion, or “waves”, of the COVID-19 pandemic. By the end of the study period, 4,244,872 Italians had been infected since the start of the pandemic.

We provide a descriptive analysis of the infections that occurred in the Veneto Region’s schools. The population tested included all students (from nursery to high school), professors, and school staff working or studying in the region during the study period. This amounted to 819,437 individuals out of a regional population estimated at 4.9 million in 2021.

### 2.1. COVID-19 Surveillance at School

**Figure 1:**
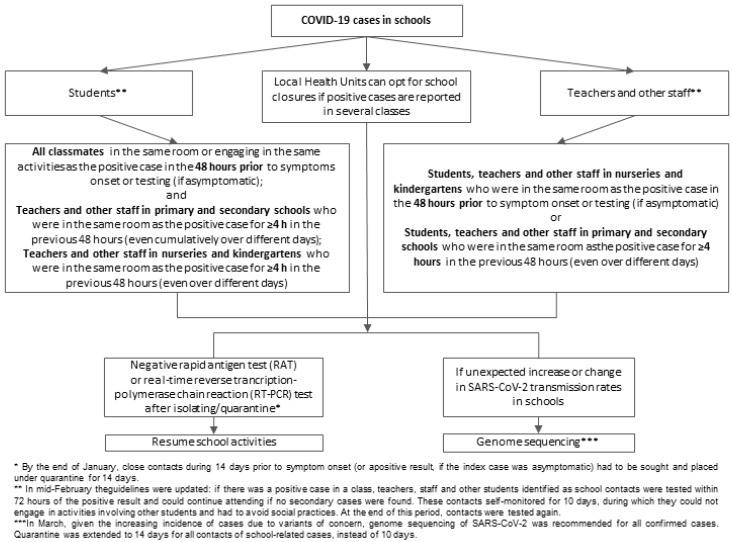
Guidelines for identifying close contacts at school.

We named and recorded as “school episodes” all cases of COVID-19 that prompted contact-tracing procedures in schools, based on the guidelines in Figure 1. The definition of confirmed cases [14] and other useful definitions are present in the Supplementary Materials.

### 2.2. Public Health Interventions at Schools in Italy and the Veneto

The Italian government allowed schools to reopen on 7 January 2021, after a national lockdown over the Christmas period. It imposed national guidelines for in-person attendance of classes to be based on the regional incidence of the infection and other epidemiological parameters. Regional authorities could adopt more restrictive measures of their own. Table 1 shows the chronological flow of the in-person attendance of classes.

### 2.3. Contact Tracing and Quarantine in Veneto Schools

All schools appointed a COVID-19 manager trained on the protocols for dealing with COVID-19 cases at school. This school COVID-19 manager coordinated hygiene measures and communicated with local public health authorities.

At the beginning of the school year, the Veneto Regional Authority established guidelines for cases of COVID-19 identified or suspected in the various types of school. The guidelines explained how contact tracing was to be performed, and established specific hygiene measures for children under six years old (e.g., social distancing arrangements, but no mask wearing due to their inability to do so properly) [8,15].

According to these guidelines, whenever a case of COVID-19 at school was confirmed (primary, middle, and high school), the public health authorities drew up a list of all close contacts with the school COVID-19 manager’s help, following a set of specific instructions (Table 1), and the individuals involved were tested within 72 h of the positive case being identified. The public health authority assessed the risk and could opt for quarantine measures or school attendance with self-monitoring for symptoms, as follows:

(a) quarantine was imposed if a positive case was identified in a nursery/kindergarten, or if there was more than one positive case in a group/class in any school year, with children self-isolating at home and being tested before returning to school;

(b) in primary, middle, and high school, if all contacts initially tested were negative, they continued to attend school, self-monitoring for symptoms, and taking another test; there were also specific recommendations for schools (e.g., avoid singing lessons, smaller classes and groups) and families (e.g., avoid extra-curricular contacts, avoid touching eyes, nose and mouth, frequent hand hygiene, avoid unnecessary travelling, and routine use of face masks). The local health authorities could also issue stricter public health requirements in specific situations, or in the event of clusters of infection.

A computerized reporting system for COVID-19 surveillance was used to routinely record attendance of school students, teachers, and staff. A specific procedure was used to monitor close contacts at school (identified by the school COVID-19 manager) by linking them all to the index case. This enabled a precise matching between the contacts listed and the individuals filed in the regional registry of cases. Each list of school contacts was also linked to a School ID code, so that the public health authorities could manage and monitor the situation to identify and link secondary cases. For all cases found positive on real-time reverse transcription-polymerase chain reaction (RT-PCR) or rapid antigen testing (RAT) and diagnosed from 4 January to 13 June, we checked whether the subject was linked to the school setting in order to include all students and educators in the surveillance.

**Figure 2:**
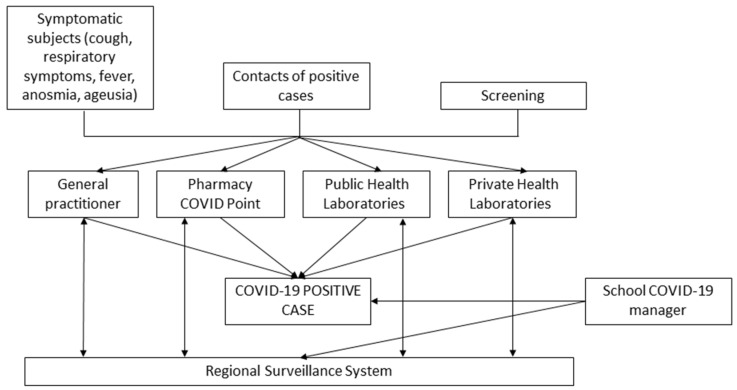
Summary of the school surveillance system.

This centralized surveillance system (Figure 2) was initially created to monitor the ongoing epidemic trends. It proved useful for tracking COVID-19 diffusion pathways, for the purposes of both epidemiological analyses and the adoption of public health measures. Data were gathered using a web-based application for recording reports of SARS-CoV-2 tests performed at all laboratories in the region (by general practitioners, hospitals, and public or private laboratories). Clinicians and public health professionals could also access this web-based application to retrieve laboratory results, and enter data on the exposure history, clinical conditions, and hospitalization history of people infected. Contact tracing and all activities related to clinical surveillance, isolation, and quarantine were managed using the same application, which allowed for the uploading of contact lists, and the creation of links between cases and their contacts.

### 2.4. Statistical Analyses

We performed proportion tests and logistic regressions on data obtained from the regional web-based application to identify differences between the types of school and index cases. The STATA 14 statistical software and Microsoft Excel were used for these analyses.

## 3. Results

**Figure 3:**
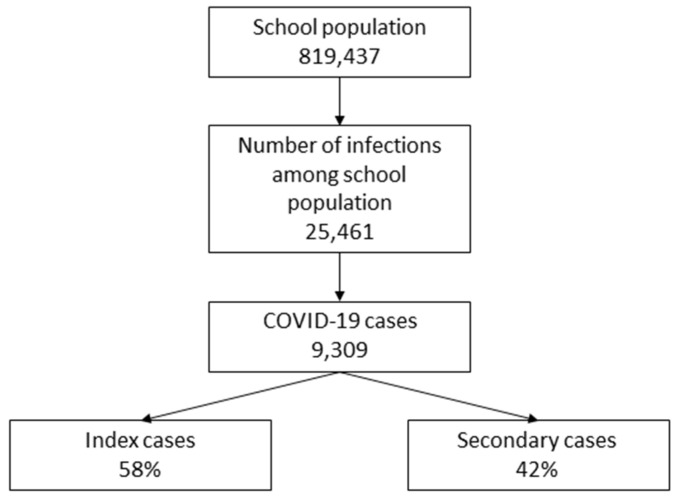
Cases of COVID-19 infection occurring at school.

Among a school population of 819,437, there were 25,418 cases identified as positive for SARS-CoV-2, whose infections occurred while they were attending school from January to June 2021 (weeks 1 to 23). School COVID-19 managers and contact tracing units described only 9309 infections as true school episodes, and only 3871 of them were secondary cases relating to index cases found at school (Figure 3).

**Figure 4:**
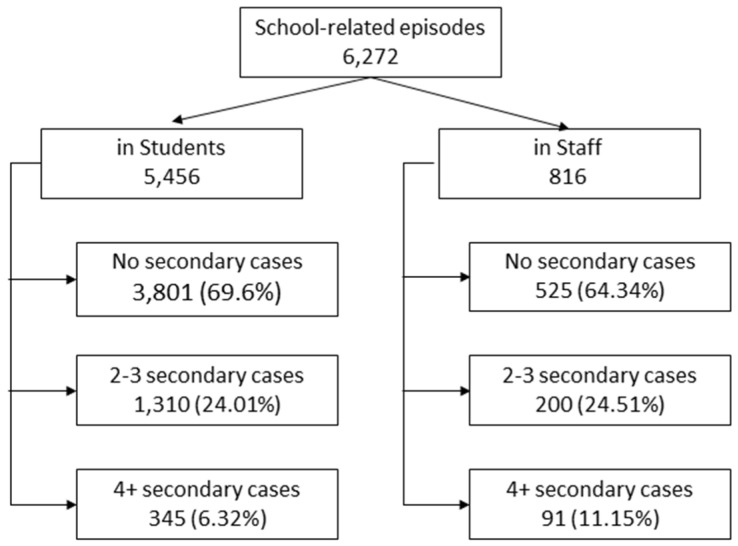
School-related episodes of infection, in students and staff.

We considered as school-related episodes all COVID-19 cases that prompted contact tracing in school settings. We identified 6272 such episodes in total, 5456 involving students, and 816 involving school staff (Figure 4).

### 3.1. Trend of School-Related Episodes

**Figure 5:**
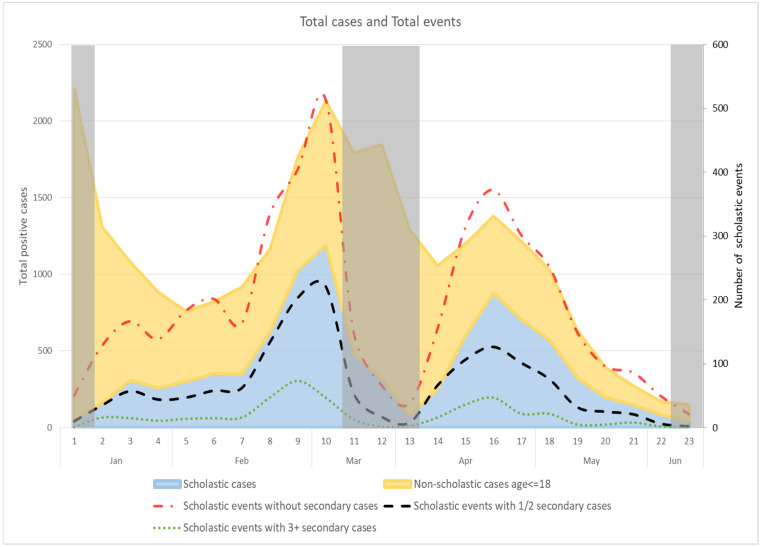
Total cases found positive in the school population (overall and school-related). Gray areas represent periods when schools were closed.

The trends of the school-related episodes and the numbers of infections occurring in the school population outside school settings are in line with the regional epidemiological trend (Figure 5). There were 25,418 infections registered in the population aged between 0 and 18 years old during the study period.

In the first week of January, the number of COVID-19 cases reported among students aged 14–18 increased. In March, after the reopening of all schools, the number of cases in the student population (all ages) rose. During the third wave, there was a similar increase in both the school population and the general population, without any anticipation of contagions.

### 3.2. Secondary Infections

Of all 6272 school-related episodes of infection that prompted the intervention of a public health operator, 31% generated secondary cases, while only 6.95% generated a cluster of three or more cases. There were no differences between the rates of infection by type of school, except for a higher percentage of secondary infections in kindergartens (*p* 0.029). Kindergartens also generated more clusters. The median number of secondary cases for each type of school was one. The results are shown in Table 2.

**Figure 6:**
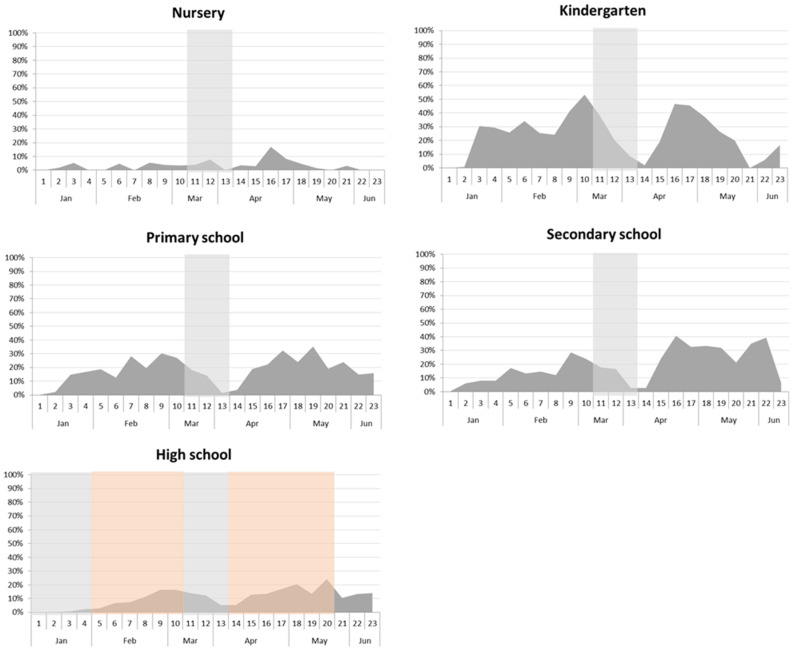
Scholastic cases (secondary cases) out of total infections by age, and by type of school. Gray areas represent periods of school closure; red areas represent those with 50–75% of in-person attendance.

We also considered the number of secondary cases related to all the positive subjects (Figure 6). Then, considering the index cases as unrelated to school settings and the secondary infections they caused as real school-related cases, our data indicate that such secondary infections accounted for 15% of all infections in the school-aged population (0–18 years old) during the first half of 2021. The percentages of scholastic cases out of total infections, by age and by type of school, were: nursery 4%; kindergarten 29.1%; primary school 17.6%; secondary school 17.8%; and high school 9.9%.

### 3.3. Index Cases

We also analyzed the data by type of school and type of index case (student or member of staff) (Table 3). As shown in Figure 4, 87% of the 6272 school-related episodes of infection involved students, and 13% involved a member of staff (teacher or educator). There was little difference in the number of secondary cases generated between the two types of index case, with a slightly higher percentage of clusters generated by staff members. The difference was greater on stratifying by type of school.

When we tested the distribution of secondary cases generated (two or more) by type of index case in kindergartens and high schools, the differences were not statistically significant. When we compared nurseries and primary schools, we found a higher proportion of episodes generated by school staff (*p* 0.0007 and 0.005). In middle schools the proportion of episodes generated by students was higher (*p* 0.004).

Merging the types of school managed under the same Veneto Regional Authority protocol, in nurseries plus kindergartens, 34% of the school-related episodes generated by a student caused secondary cases, as opposed to 42.1% of the episodes involving school staff (*p* 0.007). If we sum primary, middle, and high school secondary infections, the corresponding percentages were, respectively, 29.7% and 30.7% (*p* 0.67). We obtained the same results with a logistic model combining the type of school and type of index case with the probability of generating a secondary case: the risk was higher for school staff and early years of schooling (nursery and kindergarten).

## 4. Discussion

Our study reports on the data registered by the pandemic surveillance system used in Veneto’s schools. Thanks to the hard work of the schools’ COVID-19 managers and the contact tracing units, we can shed light on the efforts to contain SARS-CoV-2 infections in schools, hitherto assumed to be an important source of infections for society at large.

The study period covered different epidemiological trends and different national and regional provisions for schools. In the months under analysis, Italy registered second and third waves of the pandemic, reaching a total of 4,244,872 Italians infected by 13 June 2021. We considered only the school-related infections and examined the impact of schools reopening after a strict lockdown over the Christmas period. As shown in Figure 1, most of the infections in the Veneto were not linked to the school setting: 36.6% of infections occurring in the school population were school-related episodes, and only 15.2% were school-related secondary cases.

Previous international studies have tried to establish the impact of school closures on infection curves in the general population, but have so far been unable to clarify the effectiveness of school closures. They report different findings, probably due to differences in the local use of other NPIs, and in the surveillance systems adopted. In the California Bay Area experience, for instance, school closures may have reduced the number of physical interactions among students, and this could have lowered the risk of in-school transmission [16]. A significant decline in COVID-19 incidence and mortality was associated with another statewide school closure in the USA [17]. A study conducted in Israel found a sharp rise in the COVID-19 transmission rate after schools reopened [18]. On the other hand, a Japanese study found no such effectiveness of school closures [19], and German and Korean studies [20,21] came to the same conclusions. We also found no clear correlation between a rising incidence of infections in the general population and the reopening of schools. Our data suggest that higher levels of community transmission coincide with more in-school transmission because students are part of society at large, and this is particularly true in high school. It is interesting that school outbreaks did not cause any early rise in infections among the general population. In short, our findings go against the previously held assumptions that supported the closure of schools as a preventive measure in 194 countries worldwide. It is also worth noting that the rate of contagion in younger age groups remained almost constant throughout our study period. This is interesting, because nurseries and kindergartens always remained open (except over the Easter holidays), and children in these schools were not required to wear face masks or comply with social distancing rules.

To sum up, effective and accurate monitoring systems should be available, and the information collected should be unequivocal to avoid policymakers misunderstanding the day-by-day evolution of the pandemic. For monitoring systems to be efficient demands a strong cross-sectional collaboration on the part of school administrators, public health workers, general practitioners, pediatricians, and citizens. The Health in all policies approach to solving problems is, as always, the best solution, but also the most difficult to achieve. If the system cooperates, new outbreaks of SARS-CoV-2 or other pathogens can be managed more easily and faster, with less social damage caused by generalized closures. This is the only way to be better prepared for similar or even very different scenarios in the future.

In the interpretation of data, it is important to note that in high-school there was a 50–75% in-person attendance during almost the entire period of this study, while the other types of school had a 100% in-person attendance. Moreover, it is necessary to point out that any secondary case found during contact tracing in the school setting could be caused by an extra-scholastic contact (for example, during sport and other activities).

Another interesting element worth discussing is the role of vaccination in the epidemiological description of patterns of infection. Vaccine-induced immunity should now be considered as a variable in studies like ours. In the period under study here, school staff were still not fully vaccinated (1.1% fully vaccinated as of 13 March, 25.5% as 13 May), since the Italian government only identified teachers as a priority for vaccination on 10 March [22]. The vaccination program scheduled a second dose three months after the first, so our cohort was either unvaccinated, or had received only one dose. This is a limitation of our present work. On the other hand, a strength of our study lies in that the Veneto Regional Authority was able to collect real-time data at the end of the second and third waves of infection, when almost all countries were still concentrating on managing the emergency. The data we collected can serve as a useful starting point for local and national policy-makers’ evidence-based assessments, and as a baseline for other research, on the efficacy of vaccination, for instance, or the diffusion of variants of concern, and different organization models or the role of extra-scholastic activities among school infections.

## 5. Conclusions

Good practice in public health management demands tools that enable a real-time interpretation of phenomena so that they can be approached on the strength of sound evidence. Surveillance data should be easily accessible to policymakers. In any future waves of infection, they should have a clearer picture of the situation, considering all the different factors and the newly available evidence regarding the consequences of school closures.

## Figures and Tables

**Table 1:** School Calendar in the Veneto Region.

Type of School	Age	7 Janurary 2021 ^1^ 31 Janurary 2021	1 February 2021 6 March 2021	8 March 2021 13 March 2021 ^2^	15 March 2021 6 April 2021	7 April 2021 24 May 2021	26 May 2021 8 June 2021
Nursery	0–2	100% In-Person attendance			Closed	100% In-Person attendance	
Kindergarten	3–5	100% In-Person attendance			Closed	100% In-Person attendance	
Primary school	6–10	100% In-Person attendance			100% Remote Learning	100% In-Person attendance	
Middle School	11–13	100% In-Person attendance			100% Remote Learning	100% In-Person attendance	
High school	14–19	100% Remote Learning	50% In-Person attendance	50–75% In-Person attendance	100% Remote Learning	50–75% In-Person attendance	Minimum 70% In-Person attendance; 1st and 5th year students 100%

^1^ The surveillance systems began collecting records on January 4, but schools only reopened on 7 January 2021. ^2^ From March 10, attendance was suspended locally for all high schools and the second and third years of middle schools (for 12 and 13-year-olds) if the local cumulative weekly incidence of infections exceeded 250 per 100,000 population, in accordance with national legislation.

**Table 2:** Distribution of school-related secondary cases by type of school.

Type of School	No Secondary Cases	Secondary Cases	1 or 2 Secondary Cases	3 or More Secondary Cases	Mean of Secondary Cases (SD)	Median of Secondary Cases (5p–95p)
Nursery	70.5%	29.5%	21.5%	8.0%	2.15 (2.14)	1 (1–5)
Kindergarten	62.4%	37.6%	26.0%	11.7%	2.55 (2.83)	1 (1–9)
Primary school	70.4%	29.6%	23.3%	6.3%	2.16 (2.36)	1 (1–6)
Middle school	69.0%	31.0%	24.5%	6.5%	2.04 (1.80)	1 (1–6)
High school	70.9%	29.1%	24.0%	5.1%	1.83 (1.57)	1 (1–5)
Total	69.0%	31.0%	24.1%	7.0%	2.13 (2.19)	1 (1–6)

**Table 3:** Distribution of secondary cases by type of school and type of index case. The total number of infections is reported in Table S1 (Supplementary Materials).

	Index Case: Student			Index Case: Educator or Staff		
Type of School	No Secondary Cases	1 or 2 Secondary Cases	3 or More Secondary Cases	No Secondary Cases	1 or 2 Secondary Cases	3 or More Secondary Cases
Nursery	77.2%	17.9%	4.8%	52.7%	30.9%	16.7%
Kindergarten	63.8%	25.9%	10.3%	58.8%	26.1%	15.0%
Primary school	71.5%	22.9%	5.6%	62.9%	25.8%	11.3%
Middle School	68.1%	25.2%	6.7%	83.0%	13.6%	3.4%
High school	70.8%	24.0%	5.2%	74.2%	22.5%	3.4%
Total	69.7%	24.0%	6.3%	64.3%	24.5%	11.2%

## Data Availability

The datasets analyzed for the present study are not publicly available but are available from the corresponding author on reasonable request.

## Supplementary Materials

The following are available online at https://www.mdpi.com/article/10.3390/ijerph182212165/s1, Table S1: Summary table and definitions.
