# Supplementary material for: Surveillance of Outbreaks of SARS-CoV-2 Infections at School in the Veneto Region: Methods and Results of the Public Health Response during the Second and Third Waves of the Pandemic between January and June 2021
Source: Int J Environ Res Public Health. 2021 Nov 19;18(22):12165. doi: 10.3390/ijerph182212165 (PMC8624233; doi:10.3390/ijerph182212165)
Supplement: Supplementary file 1 [file ijerph-18-12165-s001.zip › ijerph-1426501-supplementary.pdf]

Supplementary material

Table S1. Summary table and definitions

	**School Grade/level**	**Nursery**	**Preschool**	**Primary school**	**Middle School**	**High school**	**Other**	**Total**
	**Student Age**	**0-2**	**3-5**	**6-10**	**11-13**	**14-18**	**-**	**-**
Scholastic Events	Number of Events	200 - (3%)	1055 - (16%)	1932 - (30%)	1401 - (22%)	1624 - (25%)	60 - (0%)	6272 - (100%)
	Index case: student	145 - (72%)	749 - (70%)	1676 - (86%)	1313 - (93%)	1535 - (94%)	38 - (63%)	5456 - (86%)
	Secondary cases genereted: No	141 - (70%)	658 - (62%)	1360 - (70%)	967 - (69%)	1152 - (70%)	48 - (80%)	4326 - (68%)
	Secondary cases genereted: 1-2	43 - (21%)	274 - (25%)	450 - (23%)	343 - (24%)	389 - (23%)	11 - (18%)	1510 - (24%)
	Secondary cases genereted: >=3	16 - (8%)	123 - (11%)	122 - (6%)	91 - (6%)	83 - (5%)	1 - (1%)	436 - (6%)
School staff	Confirmed cases	76 - (8%)	345 - (38%)	268 - (29%)	88 - (9%)	95 - (10%)	32 - (3%)	904 - (100%)
	Quarantine	495 - (8%)	2040 - (33%)	1778 - (29%)	715 - (11%)	947 - (15%)	154 - (2%)	6129 - (100%)
	School attendance with self monitoring	7 - (0%)	121 - (5%)	1119 - (50%)	451 - (20%)	479 - (21%)	52 - (2%)	2229 - (100%)
Students	Population*	105598 - (12%)	118043 - (18%)	222031 - (30%)	141385 - (23%)	232380 - (24%)		819437 - (100%)
	School available places estimated	29051 - (4%)	73518 - (10%)	222031 - (31%)	141385 - (20%)	232380 - (33%)		698365 - (100%)
	Quarantine	2563 - (3%)	18954 - (25%)	20599 - (28%)	16303 - (22%)	14813 - (20%)	143 - (0%)	73375 - (100%)
	School attendance with self monitoring	0 - (0%)	0 - (0%)	12763 - (34%)	10161 - (27%)	13456 - (36%)	233 - (0%)	36613 - (100%)
	Positive cases	2324 - (9%)	3078 - (12%)	6583 - (25%)	4892 - (19%)	8541 - (33%)		25418 - (100%)
	No School related	2086 - (89%)	1433 - (46%)	3753 - (57%)	2715 - (55%)	6165 - (72%)		16152 - (63%)
	School index cases	145 - (6%)	748 - (24%)	1673 - (25%)	1307 - (26%)	1527 - (17%)	38	5438 - (21%)
	School secondary cases	93 - (4%)	897 - (29%)	1157 - (17%)	870 - (17%)	849 - (9%)	5	3871 - (15%)

Definitions:

Confirmed case of COVID-19: a subject tested positive to RT-PCR, independently of the presence of symptoms, or to RAT (Rapid Antigen Test) in presence of symptoms or epidemiological link to COVID-19.

Secondary case: A positive case linked to the index case (for example schoolmates).

Viral clearance: the presence of one negative SARS-CoV-2 test performed at the end of the isolation period.

Contact of confirmed cases: "close" contact (high-risk exposure) and "occasional" contact (low-risk exposure) according to the definitions provided by the European Centre for Disease Prevention and Control [14].

School contact: a contact identified through criteria reported in Table 1.

School episode: the COVID-19 case that needed a contact tracing inside a school setting.
